# Jabuticaba (*Myrciaria jaboticaba*) Peel as a Sustainable Source of Anthocyanins and Ellagitannins Delivered by Phospholipid Vesicles for Alleviating Oxidative Stress in Human Keratinocytes

**DOI:** 10.3390/molecules26216697

**Published:** 2021-11-05

**Authors:** Ines Castangia, Maria Letizia Manca, Mohamad Allaw, Jarkko Hellström, Daniel Granato, Maria Manconi

**Affiliations:** 1 Department of Scienze della Vita e dell’Ambiente, University of Cagliari, 09124 Cagliari, Italy; inescastangia@tiscali.it (I.C.); mohamad.allaw@unica.it (M.A.); manconi@unica.it (M.M.); 2 Food Processing and Quality, Natural Resources Institute Finland (Luke), FI-31600 Jokioinen, Finland; jarkko.hellstrom@luke.fi; 3 Department of Biological Sciences, Faculty of Science and Engineering, University of Limerick, V94 T9PX Limerick, Ireland

**Keywords:** reactive oxygen species, circular economy, bioactives, phenolic compounds, phospholipid vesicles, wound healing

## Abstract

The Brazilian berry scientifically known as jabuticaba is a fruit covered by a dark purple peel that is still rich in bioactives, especially polyphenols. Considering that, this work was aimed at obtaining an extract from the peel of jabuticaba fruits, identifying its main components, loading it in phospholipid vesicles specifically tailored for skin delivery and evaluating their biological efficacy. The extract was obtained by pressurized hot water extraction (PHWE), which is considered an easy and low dissipative method, and it was rich in polyphenolic compounds, especially flavonoids (*ortho*-diphenols and condensed tannins), anthocyanins (cyanidin 3-*O*-glucoside and delphinidin 3-*O*-glucoside) and gallic acid, which were responsible for the high antioxidant activity detected using different colorimetric methods (DPPH, FRAP, CUPRAC and metal chelation). To improve the stability and extract effectiveness, it was incorporated into ultradeformable phospholipid vesicles (transfersomes) that were modified by adding two different polymers (hydroxyethyl cellulose and sodium hyaluronate), thus obtaining HEcellulose-transfersomes and hyaluronan-transfersomes. Transfersomes without polymers were the smallest, as the addition of the polymer led to the formation of larger vesicles that were more stable in storage. The incorporation of the extract in the vesicles promoted their beneficial activities as they were capable, to a greater extent than the solution used as reference, of counteracting the toxic effect of hydrogen peroxide and even of speeding up the healing of a wound performed in a cell monolayer, especially when vesicles were enriched with polymers. Given that, polymer enriched vesicles may represent a good strategy to produce cosmetical and cosmeceutical products with beneficial properties for skin.

## 1. Introduction

Jabuticaba, a fruit known as the Brazilian berry, belongs to the *Plinia* genus, also identified as the *Myrciaria* genus. It is a black spherical berry with a thin and fragile peel and a whitish pulp that is highly sought after and consumed fresh or in transformed products by local populations. Juices, jams, jellies, vinegars, liqueurs and wines are the main products locally obtained from these fruits [1]. Wines and liquors are produced by distillation or fermentation due to the similarity of the fruit content to that of grape. The manufacturing of these products generates a large amount of waste by-products, mainly composed of the peel and seeds, which still contain valuable compounds [2]. Indeed, the dark purple peel is rich in phenolic acids, flavonols, ellagitannins, and anthocyanins, such as cyanidin and delphinidin glucosides [3,4,5]. For this reason, jabuticaba peel has been and still is used to enrich burgers, candies, jellies and other desserts [2]. Some studies have shown that the peel is still rich in phenolic compounds that are responsible for the high antioxidant activity found in vitro using different assays [6,7], antioxidant effects in humans [8] and anti-inflammatory and gut microbiota modulation in rats [9]. Additionally, the peel could be used as a natural coloring and antimicrobial agent thanks to its high polyphenolic content. Although jabuticaba peel is a rich and valuable source of phenolic compounds with alleged beneficial effects in vivo, to date there are no technological high-value added products from jabuticaba peel on the market—neither as food nor as cosmetic products. The products created using these by-products should be in perfect accordance with the circular economy concepts and the directive of the United Nations Sustainable Development Goals, which recommends ensuring sustainable consumption and production patterns (Goal 12) [10]. One way to tackle this matter is to process fruit side-streams and by-products, such as jabuticaba peel, into effective and innovative nanotechnological products. Its valorization would be beneficial for the environment, as fruit processing units would not need to burn jabuticaba peel; for consumers, as they would consume more natural products; and for food, chemical, cosmeceutical, and pharmaceutical companies, which could reduce or even eliminate the use of synthetic chemicals in their products.

Despite the high potential of the bioactive molecules contained in the jabuticaba peel, their beneficial properties are often limited because of their low stability and reduced in vivo bioavailability. To overcome these drawbacks, nanotechnological carriers such as lipid, metallic or polymeric nanoparticles, phospholipid vesicles or micelles have recently been proposed. Overall, phospholipid vesicles, thanks to their high similarity to biological membranes, are the most biocompatible and versatile of these [11,12]. Indeed, in recent decades, liposomes and other modified phospholipid vesicles have been developed and tested as carriers of synthetic drugs and natural bioactive molecules [13]. In particular, deformable vesicles, so called transfersomes, have been specifically designed for skin delivery and have sometimes been stabilized by adding hydrophilic polymers to the formulation. The latter interact with the bilayer in the internal and/or external surface, favoring sterically stabilized vesicles in dispersion [14,15,16]. Hydrophobically modified hydroxyethyl cellulose was previously used to coat liposomes, improving their stability [17]. Hydroxyethyl cellulose is a water-soluble polysaccharide derivative widely used in topical formulations. It forms a film on the skin surface, avoiding its transpiration, improving the water content and fluidity of stratum corneum, thus facilitating the passage of external molecules [18]. Sodium hyaluronate has also previously been combined with phospholipid vesicles or used alone in topical formulations due to its beneficial properties. Indeed, it is the major glycosaminoglycan contained in the extracellular matrix of most mammalian tissues, especially the dermis. It is implicated in several biological functions of the skin, thus favoring its restoration and stimulating wound healing.

In this study, the two polymers were alternatively combined with transfersomes to improve the vesicle delivery performances and stability. The resulting vesicles were used to load the extract of jabuticaba peel, which was previously obtained by pressurized hot water extraction (PHWE). The chemical composition and antioxidant activity of the extract was analyzed, along with its ability, when loaded in vesicles, to reduce oxidative damage in skin and to promote wound healing.

## 2. Results and Discussions

### 2.1. Jabuticaba Peels: Chemical Characterization and Antioxidant Activity

Mass spectrometric detection of anthocyanins in jabuticaba peel extract was primarily performed in positive mode. Two compounds with molecular ions at (*m/z*)^+^ 449.107 and 465.100 produced fragment ions of 287.054 and 303.045, respectively, and they were identified as cyanidin and delphinidin hexosides. Ionization of anthocyanins was much weaker in negative mode, but nevertheless characteristic ions at (*m/z*)^−^ 447.091 (M − 2)^−^ and 465.101 (M + H_2_O − 2)^−^ for cyanidin-hexoside and 463.085 (M − 2)^−^ and 481.095 (M + H_2_O − 2)^−^ for delphinidin-hexoside could be seen, confirming the negative ion formation mechanism proposed by Sun et al. [19] for anthocyanins. On HPLC-DAD, the two major anthocyanins in jabuticaba peel had similar retention times and UV-spectra to authentic standards of cyanidin 3-*O*-glucoside and delphinidin 3-*O*-glucoside. They represented 1.5% of the phenolic content of the gallic acid extract and total ellagitannins accounted for 4.1% and 12.7% of the total phenolic content. These results are similar to those reported in jabuticaba seeds from different varieties [9,20].

The phenolic components of the extract were identified and the antioxidant activity was measured using different assays (Table 1). The lyophilized jabuticaba peel extract contained 7.1% of the total phenolic compounds, in which flavonoids, *ortho*-diphenols and condensed tannins were 26.4%, 11.1% and 7.0% of the total phenolic composition, respectively.

**Figure 1:**
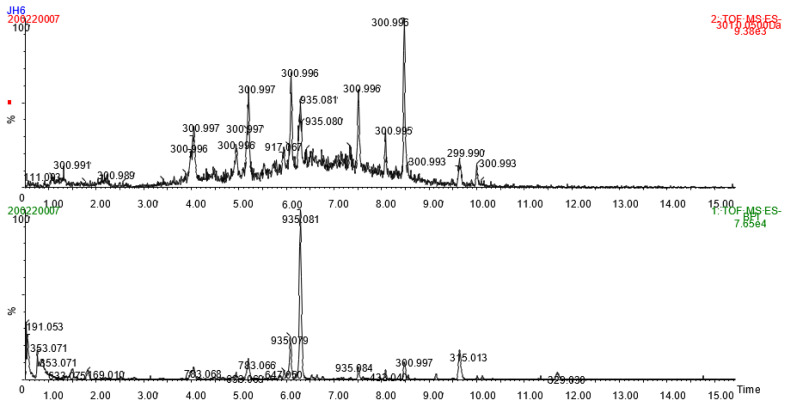
Base peak intensity chromatogram and MS^E^ chromatogram at *m/z* 301.0 for jabuticaba peel extract.

Figure 1 shows the base peak intensity chromatogram and MS^E^ chromatogram at *m/z* 301.0 for jabuticaba peel extract in negative mode. Deprotonated ellagic acid (*m/z* 301.0) is a typical fragment ion of ellagitannins. Several peaks in the MS^E^ chromatogram (Figure 1) indicate the presence of various ellagitannins eluting generally during the first ten minutes of the UHPLC run. MS data of identified ellagitannins is shown in Table 2. Gallolyated and non-gallolyated hexahydroxydiphenic acid (HHDP) glycosides, namely galloyl-HHDP-hexoside bis-HHDP-hexoside (three isomers), castalagin/vescalagin (two isomers), trigalloyl-HHDP-hexoside, digalloyl-HHDP-hexoside, galloyl-bis-HHDP-hexoside (three isomers), and ellagic acid pentoside were identified in accordance with previous studies of jabuticaba [21,22,23,24]. A precursor ion at *m/z* 1067.1189 (Rt 5.86 min) suggested a deprotonated ion of the compound with a molecular formula of C_46_H_36_O_30_ and it was tentatively identified as pterocarinin A due to its similar fragmentation pattern (Table 2) to that reported previously for pterocarinin A [25]. A double-charged ion at *m/z* 858.0645 was detected (Rt 7.36 min) with fragment ions common for ellagitannins (Table 2). Assuming typical (M − 2H)^2−^ ion formation, the double-charged ion could originate from a compound with a molecular formula of C_75_H_50_O_48_. The formula would match that of degallolyated sanguiin H-6, an ellagitannin that has been frequently detected in fruits of the *Rubus* family [26,27]. However, further research is needed to fully characterize the compound.

The lyophilized jabuticaba peel extract scavenged free radicals, reducing power and metal chelating properties, thanks to its phenolic content that is known to counteract oxidative stress not only in vitro but also in vivo [9,21,28]. Thus, considering the abundance and profile of phenolic compounds in the lyophilized aqueous extracts of jabuticaba peel, their antioxidant potential was measured by different assays and standard molecules that were used as reference (Table 1).

### 2.2. Preparation and Characterization of the Phospholipid Vesicles

Considering the rich phenolic content of the jabuticaba peel extract and the low bioavailability of these molecules [29,30], especially on the skin, it was loaded into transfersomes—phospholipid vesicles tailored for topical application [31,32]. To this end, an edge activator (Tween 80) was added to the phospholipids to increase the bilayer fluidity and the vesicle ability to squeeze through the inter-corneocyte matrix, increasing the payload deposition in the deeper skin layers [31]. Indeed, previous studies confirmed that this surfactant added to the phospholipid vesicles promoted their skin delivery performance [32,33]. In addition, transfersomes were enriched with hydroxyethyl cellulose and hyaluronic acid to promote vesicle stability. Then, a natural polymer (hyaluronic acid) and a semi-synthetic one (hydroxyethyl cellulose) were used and their effect on vesicle stability was compared. In previous studies, it was demonstrated that hyaluronic acid associated with phospholipids and formed optimal vesicles, called hyalurosomes, where hyaluronan is distributed on the internal and external vesicle surface, favoring their stability along with their skin delivery aptitude [13]. Hydroxyethyl cellulose is a semi-synthetic polymer widely used for topical formulation due to its thickening and gelling properties [34]. It is considered a safe and biocompatible ingredient capable of increasing the spreadability of the system and its application on the skin. Despite its promising properties for topical applications, its actual interaction with phospholipid vesicles has not previously been studied [35]. In this study, both polymers were added at two different concentrations: 1 and 2 mg/mL (Table 3).

The average diameter, polydispersity index and zeta potential of the transfersomes were measured (Table 4). The transfersomes were the smallest vesicles with a mean diameter of around 62 nm (*p* < 0.05 versus the mean diameter of other vesicles) and a low polydispersity index (0.23). The addition, polymers caused a slight increase of the mean diameter (around 91 nm, *p* > 0.05 among the mean diameter of vesicles) irrespective of the polymers and concentrations used. All vesicles were negatively charged (~−19 mV) without significant differences between samples, probably because the amount of both polymers was very low (1 or 2 mg/mL) in comparison with the amount of phospholipid used (180 mg/mL). Thus, it could not significantly affect the Z-potential of the vesicles.

**Figure 2:**
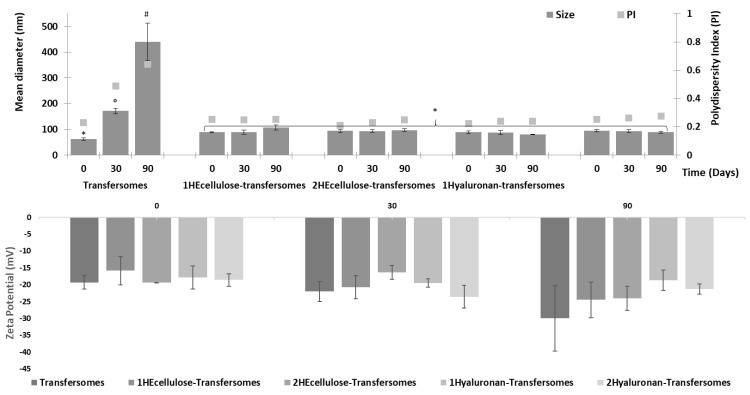
Mean diameter (MD), polydispersity index (PI) and zeta potential of extract loaded transfersomes stored for 90 days at room temperature (~25 °C). Mean values (bars) ± standard deviations (error bars) are reported (*n* = 3). Each symbol indicates the same value that is statistically different from those indicated by the other symbols (*p* < 0.05).

All the formulations incorporated high amounts of jabuticaba peel extract (Table 4). Indeed, the incorporation efficiency was always higher than 90% without significant differences between the different samples, confirming that the addition of the polymers did not modify the ability of the vesicles to incorporate and retain the phytochemicals contained in the extract. The high encapsulation efficiency may be due to the antioxidant molecules mainly being located in the vesicle bilayer, which is not released during the purification process. In addition, the high amount of phospholipid used (180 mg/mL) led to the formation of a large number of vesicles, which in turn increased the viscosity of the dispersion—thus reducing the mobility of both the vesicles and the molecules. The stability of the vesicles was assessed by storing them at room temperature (~25 °C) for 90 days and measuring their main physicochemical properties (size, polydispersity index and zeta potential) at scheduled times (Figure 2). The mean diameter of transfersomes, which were initially the smallest, increased up to around 180 nm at 30 days and up to 450 nm at 90 days. The polidispersity index increased as well, and the samples appeared to be biphasic. On the contrary, the transfersomes enriched with the polymers maintained the same characteristics, mean diameter (~91 nm), polydispersity (~0.24) and zeta potential (~−20 mV). The improvement of the stability was not affected by the concentration or type of polymer [36]. Our results showed that the polymer addition improved vesicle stability in the dispersion, probably immobilizing them in the polymeric network.

### 2.3. In Vitro Studies with Keratinocytes

#### 2.3.1. Biocompatibility of Vesicles

**Figure 3:**
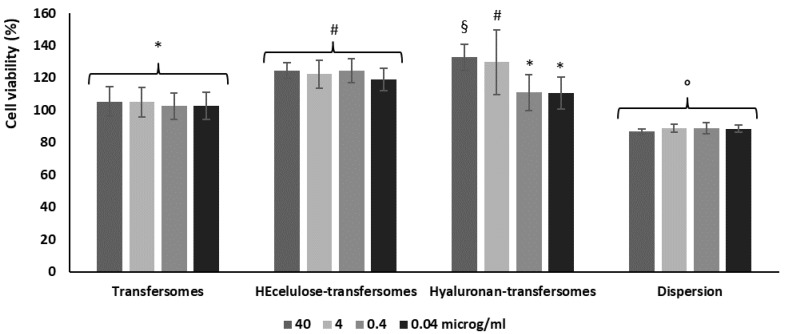
The viability of keratinocytes following treatment for 48 h with the extract in dispersion or incorporated into transfersomes. Mean values (bars) ± standard deviations (error bars) are reported (*n* = 8). Each symbol indicates the same value that is statistically different from those labelled with other symbols (*p* < 0.05).

The study performed to evaluate the physicochemical properties of transfersomes, especially those enriched with the polymers, confirmed their greater stability and ideal size to be applied on the skin. Any important difference was detected as a function of the polymer concentrations (1 or 2 mg/mL). In order to assess this, the vesicles modified with the highest polymer concentration (2 mg/mL) were used to perform the subsequent studies. Their biocompatibility was evaluated using keratinocytes and that of the extract in aqueous dispersion was evaluated as well and used as a comparison. Keratinocytes have been chosen as they are the most representative cells of the epidermis and a layer-by-layer setup was used since in their special differentiation they form the main barrier of the skin, which regulates skin hydration and prevents exogenous substances from penetrating into and through it. Keratinocytes were treated for 48 h with the extract in dispersion or incorporated within the vesicles at four different dilutions, after which the cell viability was measured (Figure 3). The viability of cells incubated with the extract in dispersion was ~88% (*p* < 0.05 versus the viability measured using extract loaded vesicles), indicating that the extract is not toxic. The viability of cells treated with extract-loaded transfersomes and hyaluronan-transfersomes at higher dilutions was ~100% (*p* > 0.05 among this group). The treatment with HEcellulose-transfersomes and hyaluronan-transfersomes further improved cell viability. All formulations were highly biocompatible regardless of the polymer and dilution used. Indeed, the cell viability was equal to or higher than 100%, even showcasing a proliferative effect associated with vesicle loading.

#### 2.3.2. Protective Effect of the Extract, in Dispersion or Loaded in Vesicles, against Damage Induced in Keratinocytes by Hydrogen Peroxide

**Figure 4:**
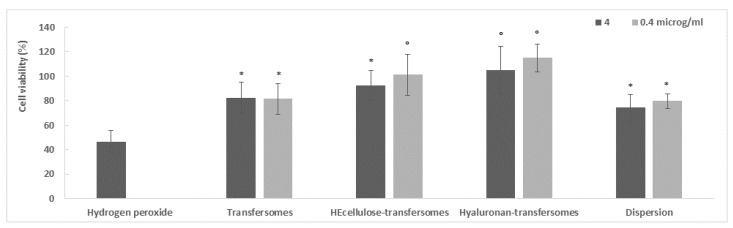
Viability of keratinocytes stressed with hydrogen peroxide and protected with the extract in dispersion or incorporated in transfersomes. Mean values (bars) ± standard deviations (error bars) are reported (*n* = 8). Each symbol indicates the same value and is statistically different from those labeled with other symbols (*p* < 0.05).

The keratinocytes were stressed with hydrogen peroxide and then treated with the extract either in dispersion or loaded into transfersomes. The cell viability was measured at 4 h and this was used to evaluate the protective effect of the formulations against oxidative stress (Figure 4). The samples were diluted with the cell medium to reach two different concentrations (4 and 0.4 μg/mL). The hydrogen peroxide stress caused high cell mortality and reduced viability by up to ~50% (*p* < 0.05 versus the viability of cells treated with the extract in dispersion or loaded in vesicles) [35]. Hydrogen peroxide is considered one of the most dangerous oxidative molecules among the different reactive oxygen species, capable of promoting apoptosis and cell death. The treatment of stressed cells with the aqueous dispersion of the extract was capable of reducing the damaging effect of hydrogen peroxide as the viability increased up to ~78% (*p* < 0.05 versus the viability of cells treated with the extract loaded in HEcelluose-transfersomes at a higher dilution and hyaluronan-transfersomes), although the complete restoration of normal conditions was not achieved (Figure 4). Treatment with the extract loaded transfersomes protected the cell to the same extent as the extract in dispersion (~82%, *p* > 0.05 versus the viability of cells treated with dispersion). The treatment with the best result was the extract loaded with HEcellulose-transfersomes (at a higher dilution) and hyluronan-transfersomes, which achieved a viability of ~104% (*p* < 0.05 versus the values of other treatments). They restored normal conditions and even slightly promoted cell proliferation, probably due to the synergic effect of the extract and the polymer-immobilized vesicles. The vesicle behavior was not affected by the dilution levels of the samples.

#### 2.3.3. In Vitro Wound Healing Effects

**Figure 5:**
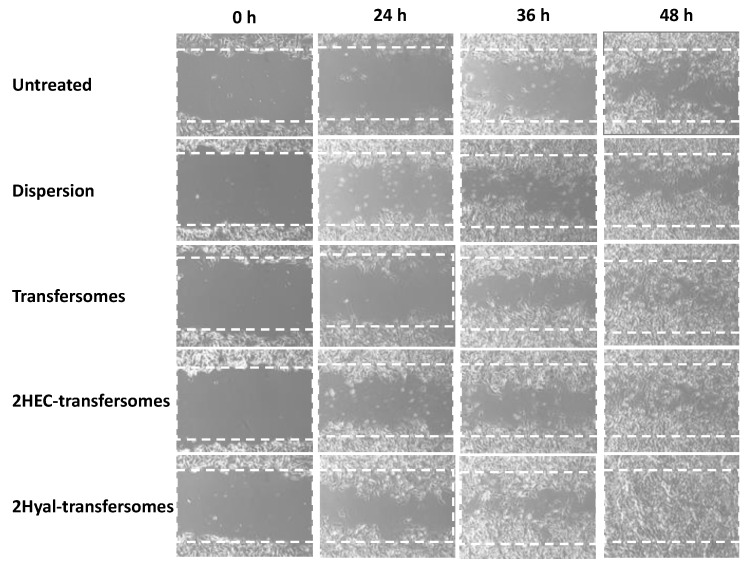
Representative images of lesions performed on a keratinocyte monolayer untreated or treated with the extract in a water dispersion or loaded into transfersomes.

**Figure 6:**
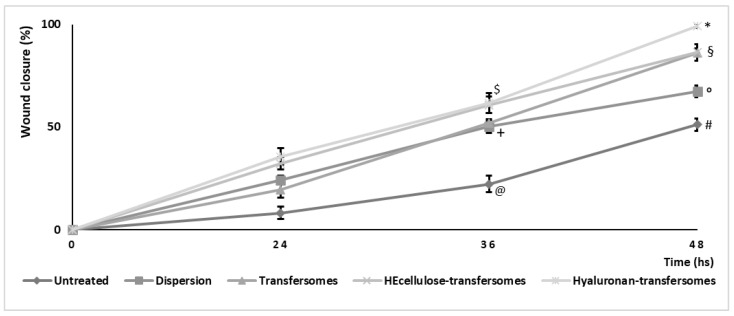
Wound closure (%) measured in a keratinocyte monolayer untreated or treated with the extract in water dispersion or loaded in transfersomes. Mean values ± standard deviations (*n* = 3) are reported. Each symbol indicates the same value and is statistically different from the values labeled with other symbols (*p* < 0.05).

The in vitro scratch assay was performed with a monolayer of keratinocytes in order to verify the ability of the extract in aqueous dispersion or incorporated into vesicles to stimulate the proliferation and migration of cells. Empty vesicles were not tested as in a previous study no improvement in wound healing was detected when empty hyalurosomes were used [37]. The closure of the performed wound was monitored for 48 h (Figure 5) and the % of closure was calculated by measuring the lesion areas (Figure 6). The wound closure of untreated cells occurred very slowly—13% at 24 h, 28% at 36 h and only 40% at 48 h. Treatment with the extract in water dispersion slightly improved the process and bringing the closure at 48 h to 50%. Treatment with the extract loaded vesicles at 48 h achieved 90% closure when using transfersomes and HEcellulose-transfersomes and around 100% closure when using hyaluronan-transfersomes. As we can see, the wound closure was almost complete, confirming the restoring properties of the extract-loaded hyaluronan-transfersomes.

Our overall results confirmed the high biocompatibility of hyaluronan-transfersomes, which were also the most effective at stimulating the proliferation and migration of skin cells and counteracting the damage induced in skin by oxidative stress.

## 3. Materials and Methods

### 3.1. Materials

Lipoid S75 (consisting of ~70% of soy phosphatidylcholine, 9% phosphatidylethanolamine and 3% lyso-phosphatidylcholine) was purchased from Lipoid GmbH (Ludwigshafen, Germany). Sodium hyaluronate with low molecular weight (200–400 kDa) and a polydispersity of 1.4 Mw/Mn, was purchased from DSM Nutritional Products AG Branch Pentapharm (Switzerland). Tween 80, glycerol, DPPH radical (2,2-diphenyl-1-picrylhydrazyl), ferric chloride hexahydrate, gallic, chlorogenic, and ascorbic acids, catechin, tetrazolium salt, 3-(4,5-dimethylthiazol-2-yl)-2,5-diphenyltetrazolium bromide (MTT), quercetin, neocuproin, Folin–Ciocalteu’s phenol reagent, 2,4,6-tris(2-pyridyl)-S-triazine (TPTZ) and pyrocatechol violet were purchased from Sigma-Aldrich (Milan, Italy). Potassium hexacyanoferrate (III) was obtained from Merck (Darmstadt, Germany). Anhydrous sodium acetate, copper sulfate pentahydrate and copper chloride dihydrate were obtained from VWR Chemicals BDH^®^ (Frankfurt, Germany). Cyanidin 3-*O*-glucoside (≥95%) and delphinidin 3-*O*-glucoside (≥95%) were purchased from Extrasynthese (Lyon, France). Glacial acetic acid and phosphoric acid (85%) were acquired from J.T. Baker (Mallinckrodt Baker Inc., Utrecht, The Netherlands). All reagents and plastics for cell culture were purchased from Life Technologies Europe (Monza, Italy).

### 3.2. Jabuticaba Peels: Extraction, Chemical Characterization, and Antioxidant Activity

Jabuticaba fruits (*Myrciaria jabuticaba* (Vell.) O.Berg) cv. Sabará were harvested at a ripe maturation stage in Araucária city, Paraná, Brazil (geographical coordinates: 25°2924.7″ S 49°2637.8″ W) in December 2018. Fruits were washed and sanitized (NaOCl at 100 mg/L/15 min), rinsed and pulped manually. Peels were dried at 35 °C for 50 h, ground to reach 42 Tyler mesh, and extracted in an accelerated solvent extractor (ASE-350, Dionex, Sunnyvale, CA, USA), employing a pressure of 100 bar (98.7 atm) in two extraction cycles at 50 °C. Water acidified with citric acid (pH 2.10) was used as the solvent and extractions were repeated four times. Then, extracts were filtered using qualitative paper and freeze-dried under vacuum for 120 h. Following this, the extraction yield, expressed as a percentage, was calculated concerning the raw material used in the procedure.

The total phenolic content (TPC, mg of gallic acid equivalent per 100 g, mg GAE/100 g), total condensed tannin (TCT, mg of catechin equivalent per 100 g, mg CE/100 g), total flavonoids (TF, mg CE/100 g) and total *ortho*-diphenols content (TOD, mg of chlorogenic acid equivalent per 100 g, mg CAE/100 g) were measured in triplicate by using UV-Vis spectrophotometry according to the procedures fully described by do Carmo et al. [20].

Anthocyanins (cyanidin 3-*O*-glucoside and delphinidin 3-*O*-glucoside) were quantified by high-performance liquid chromatography (HPLC) using an Agilent 1100 (Agilent Technologies Inc., Espoo, Finland) device equipped with diode array detection (DAD). Separation was performed using a Gemini C_18_ column (150 mm × 4.6 mm, 5 μm) with a gradient elution of acetonitrile acidified with formic acid at 5%. Total ellagitannin content was determined after acid hydrolysis, according to Mattila and Kumpulainen [38], and gallic acid was determined using an Inertsil ODS-3 column (150 mm × 4.0 mm, 3 μm) with a gradient elution of acetonitrile into 50 mmol/L H_3_PO_4_ (pH 2.5). Analyses were performed in triplicate and results expressed as mg/100 g.

Ultra-high performance liquid chromatography (UHPLC) combined with high resolution mass spectrometry (MS) was applied for the characterization of major ellagitannins and anthocyanins. An Acquity UPLC-Xevo G2 QTOF mass spectrometer (Waters, Milford, MA, USA) was equipped with a Waters Acquity BEH C18 (1.7 µm, 2.1 mm × 150 mm) column and the separation was performed using a gradient of acetonitrile into water acidified with 0.1% formic acid, according to Santos et al. [39]. The flow rate was 0.55 mL/min, the temperature of the column oven was 45 °C and the injection volume was 1.0 µL. An electrospray interface (ESI) in negative and positive mode was used with capillary voltages of −1 kV and +0.5 kV, respectively. Argon was used as the collision gas. MS analyses were conducted by data independent acquisition (MSE) centroid data mode in a full scan *m/z* 50–1500 with 0.2 s scan time. In the MSE function, the precursor ions of MS were fragmented using high collision energy ramped up from 25 to 45 V.

The antioxidant activity of the extract was analyzed in triplicate by using different assays: free-radical scavenging activity in relation to the DPPH radical, ferric reducing antioxidant power (FRAP), cupric-ion reducing antioxidant capacity (CUPRAC), reducing power and Cu^2+^ chelating ability. Results were expressed as mg GAE/100 g (reducing power), mg of ascorbic acid equivalent per 100 g, mg AAE/100 g (DPPH, FRAP, and CUPRAC) and mg EDTA equivalents/100 g (metal chelation), respectively. All methods used have been deeply described previously by do Carmo et al. and Fidelis et al. [9,20].

### 3.3. Vesicle Preparation

Transfersomes, hydroxyethyl cellulose enriched transfersomes (HEcellulose-thansferomes) and sodium hyaluronate enriched transfersomes (hyaluronan-transfersomes) were prepared by dispersing phospholipid (S75, 180 mg/mL), Tween 80 (20 mg/mL), extract (40 mg/mL) and polymer (hydroxyethyl cellulose or sodium hyaluronate 1 and 2 mg/mL), when appropriate, in water and leaving the blends to hydrate for a few hours. Following this, the dispersions were sonicated (4 cycles 2 on 5 off 15.0 μ amplitude), waiting 5 min between each cycle to promote cooling and avoid overheating of the sample. A high performance Soniprep 150 sonicator (MSE Crowley, London, UK) was used to sonicate all dispersions in order to obtain a homogeneous system with a small size [15]. This procedure avoids the loss of the components used for the preparation, leading to the formation of performant vesicles capable of incorporating high amounts of the extract.

### 3.4. Characterization of the Vesicles

The average diameter and the polydispersity index (a dimensionless measure of the broadness of the size distribution) of the vesicles were measured by photon correlation spectroscopy using a Zetasizer Ultra (Malvern Instrument, UK). The same equipment was used to measure the zeta potential of vesicles by measuring the electrophoretic mobility of particles [40]. The samples were suitably diluted before measurement to be optically clear and avoid the reduction of scattered light that can be detected. To evaluate the amount of extract actually incorporated into the vesicles, the dispersions were purified from the non-incorporated extract by dialysis. Vesicle dispersions (1 mL) were inserted into polycarbonate dialysis tubes (Spectra/Por^®^ membranes: 12–14 kDa MW cut-off, with pores 3 nm; Spectrum Laboratories Inc., Rancho Dominguez, CA, USA) and immersed in distilled water (2 L) at 25 °C for 2 h under stirring. The water was refreshed after one hour, thus using 4 L of water to solubilize the bioactive molecules not incorporated in 1 mL of vesicle dispersion (40 mg). The amount of extract in the vesicle suspensions before and after the dialysis was quantified measuring their antioxidant activity using the DPPH assay. The entrapment efficiency of the extract inside the vesicles was calculated as a percentage ratio between the antioxidant activity of the samples before and after the purification process [41,42,43].

### 3.5. Stability of Vesicles on Storage

The stability of the vesicles in the dispersion was evaluated by monitoring their average size, the polydispersity index and the surface charge for 90 days while keeping the dispersions at room temperature (25 ± 1 °C).

### 3.6. Measurement of the Antioxidant Activity of Samples Using the DPPH Colorimetric Test

The antioxidant activity of jabuticaba peel extract loaded into vesicles was measured as a function of its ability to scavenge DPPH. The dispersions (10 μL) were diluted (1:100) with a methanolic solution of DPPH (0.4 μg/mL). The diluted samples were stored at room temperature and in the dark for 30 min, then the absorbance of the solutions was measured at 517 nm using a UV spectrophotometer. The antioxidant activity of the formulations was calculated according to Equation (1):

Antioxidant activity% = [(ABS_DPPH_ − ABS_sample_)/ABS_DPPH_] × 100 (1)

### 3.7. Biocompatibility and Protective Effect of Samples against Oxidative Stress in Keratinocytes

Immortalized human keratinocytes (HaCaT) were grown as monolayers at 37 °C, 100% humidity and 5% CO_2_ using Dulbecco’s Modified Eagle Medium (DMEM) high glucose supplemented with foetal bovine serum, penicillin and streptomycin as growth medium. To evaluate the biocompatibility of formulations, cells were seeded into 96-well plates at a density of 7.5 × 10^3^ cells/well. After 24 h, cells were treated for 48 h with jabuticaba extract in aqueous dispersion or loaded in vesicles properly diluted with DMEM to reach different concentrations of the extract (40, 4, 0.4 and 0.04 μg/mL). At the end of the incubation, MTT [3(4,5-dimethylthiazolyl-2)-2, 5-diphenyltetrazolium bromide] (100 µL, 0.5 mg/mL final concentration) was added to each well, and, after three hours, the formed formazan crystals were dissolved with dimethyl sulfoxide. The absorbance of each well was measured at 570 nm using a microplate reader (Synergy 4 Reader, BioTek Instruments, AHSI S.p.A, Bernareggio, Italy). All experiments were repeated at least three times, each time in triplicate. Results are shown as percent of cell viability in comparison with untreated control cells (100% viability).

The in vitro protective effect of formulations against damage caused by oxidative stress was evaluated as well. Cells were seeded into 96-well plates at a density of 7.5 × 10^3^ cells/well. After 24 h of incubation, cells were stressed with hydrogen peroxide (30% diluted 1:40,000 *v/v* with PBS) and treated with the extract in aqueous dispersion or loaded in vesicles and diluted to reach two different concentrations (4 and 0.4 µg/mL). Cells stressed with hydrogen peroxide only were used as negative control, while untreated cells were used as positive control. After 4 h of incubation, cells were washed with fresh medium and their viability was determined by the MTT assay. Results are reported as the percentage of untreated cells (100% viability).

### 3.8. In Vitro Wound Healing Properties

The ability of the jabuticaba peel extract loaded into vesicles to remodel the skin lesions and promote their healing was evaluated by measuring the cell expansion on a lesion in a cell monolayer. The cells were cultured in 6 well plates until a complete and homogeneous monolayer was reached. A linear wound was generated using a sterile plastic pipette tip. The scattered fragments of cells were removed by gentle washing with fresh medium. Extract aqueous dispersion or extract loaded vesicles were diluted with cell medium up to 4 μg/mL of extract and used to treat the lesioned cell monolayers. The cell lesions were observed, and the images were captured at 24, 36 and 48 h of incubation using an optical microscope with a 10× objective. Untreated cells were used as negative control. The area lesions in the captured images were measured by Java’s image J software (http://rsb.info.nih.gov, accessed in April–June 2021). The closure of the wounds was calculated using Equation (2):

wound closure (%) = [(a_0_ − a_t_/a_0_] × 100% (2)

where a_0_ is the wounded area immediately after scratching, and a_t_ is the wounded area measured at 24, 36 and 48 h [44].

### 3.9. Statistical Analysis

Results are expressed as the mean ± standard deviation and significance was tested at the 0.05 level of probability (*p*). For size, zeta potential, viscosity, drug accumulation and cytotoxicity, one-way analysis of variance (ANOVA) was used to substantiate statistical differences between groups followed by Tukey’s test, while Student’s *t*-test was used for comparison between two samples using XLStatistic for Excel.

## 4. Conclusions

The lyophilized jabuticaba peel extract was incorporated into transfersomes and polymer (hydroxyethylcellulose and sodium hyaluronate) enriched transfersomes with the aim of stabilizing the extract and improving its therapeutic efficacy. The obtained vesicles were small in size and homogeneously dispersed. The addition of both polymers only led to the formation of slightly larger vesicles without differences between the polymeric concentrations used. Polymer-enriched vesicles seemed to be ideal for topical administration and were capable of incorporating the bioactive-rich extract in high amounts. In particular, the combination of phospholipid, Tween 80 and sodium hyaluronate to obtain Hyaluronan-transfersomes, has been selected as the best formulation in terms of stability and ability to interact with keratinocytes, as only these vesicles were able to effectively counteract the damage induced in cells when using hydrogen peroxide and to promote wound-healing in human keratinocytes. Overall, our results suggested that hyaluronan-transfersomes may represent a promising system for the treatment of skin diseases or skin wounds connected with oxidative stress. In addition, we show for the first time the use of jabuticaba peel extract in a dermatological delivery system.

## Figures and Tables

**Table 1:** Phenolic compounds measured in the lyophilized jabuticaba peel extract and antioxidant activities measured by different assays referred to as gallic acid equivalent (GAE), chlorogenic acid equivalent (CAE), catechin equivalent (CE), ascorbic acid equivalent (AAE) and EDTA equivalent (EDTAE).

Phenolic Composition and Antioxidant Activity	Content
Total phenolic content	7090 ± 43 mg GAE/100 g
*ortho*-Diphenols	784 ± 3 mg CAE/100 g
Total flavonoids	1870 ± 31 mg CE/100 g
Condensed tannins	498 ± 13 mg CE/100 g
Total anthocyanins	107 ± 3 mg/100 g
Total ellagitannins content	901 ± 3 mg/100 g
Delphinidin 3-*O*-glucoside	8 ± 0.21 mg/100 g
Cyanidin 3-*O*-glucoside	61 ± 0.51 mg/100 g
Gallic acid	290 ± 6 mg/100 g
FRAP	10768 ± 232 mg AAE/100 g
DPPH	6807 ± 108 mg AAE/100 g
Reducing power	1921 ± 10 mg GAE/100 g
CUPRAC	27983 ± 393 mg AAE/100 g
Cu^2+^ chelating ability	20696 ± 172 mg EDTAE/100 g

**Table 2:** MS data on anthocyanins and ellagitannins in jabuticaba peel extract. HHDP = hexahydroxydiphenoyl acid, Hex = hexoside, Pen = pentoside.

Anthocyanins					
*Rt (min)*	*Suggested Compound*	*M+*	*Exact Mass*	*Detected Mass*	*Fragment ions*
5.84	Delphinidin-Hex	C_21_H_21_O_12_	465.1033	465.1017	303.0458
6.29	Cyanidin-Hex	C_21_H_21_O_11_	449.1084	449.1064	287.0533
**Ellagitannins**					
*Rt (min)*	*Suggested Compound*	*(M-H)-*	*Exact Mass*	*Detected Mass*	*Fragment ions*
1.35	Galloyl-HHDP-Hex	C_27_H_21_O_18_	633.0734	633.0745	481.0612, 300.9947, 275.0160
4.04	Bis-HHDP-Hex	C_34_H_23_O_22_	783.0681	783.0683	481.0623, 300.9966, 275.0177, 249.0357
4.47	Castalagin/Vescalagin	C_41_H_25_O_26_	933.0634	933.0650	915.0612, 783.0648, 425.0042, 300.9958, 275.0140, 249.0357
4.88	Castalagin/Vescalagin	C_41_H_25_O_26_	933.0634	933.0617	783.0716, 425.0010, 300.9959, 275.0163
4.94	Bis-HHDP-Hex	C_34_H_23_O_22_	783.0681	783.0643	481.0614, 300,9954, 275.0161, 249.0287
5.19	Bis-HHDP-Hex	C_34_H_23_O_22_	783.0681	783.0654	481.0569, 300.9959, 275.0514, 249.0369
5.30	Trigalloyl-HHDP-Hex	C_41_H_27_O_27_	951.0740	951.0725	907.0729, 783.0651, 481.0534, 300,9956, 275.0153, 249.343
5.86	Pterocarinin A	C_46_H_35_O_30_	1067.1213	1067.1189	1023.1228, 933.1010, 377.0256, 300.9955, 275.0174, 249.0358
6.08	Galloyl-Bis-HHDP-Hex (casuarinin)	C_41_H_27_O_26_	935.0791	935.0770	917.0676, 873.0729, 855.0708, 783.0633, 633.0699, 300.9955, 275.0170. 249.0360
7.04	Digalloyl-HHDP-Hex	C_34_H_25_O_22_	785.0837	785.0865	633.0690, 300.9957, 275.0128, 261.0000, 249.0363
7.18	Galloyl-Bis-HHDP-Hex	C_41_H_27_O_26_	935.0791	935.0787	783.0715, 633.0704, 463.0501, 300.9964, 275.0180, 249.0375
7.36	degallolyated Sanguiin H-6	C_75_H_49_O_48_	858.0660 / (M − 2H)^2−^	858.0645 / (M − 2H)^2−^	935.0776, 783.0776, 633.0709, 300.9958, 275.0175, 249.0368
7.51	Galloyl-Bis-HHDP-Hex	C_41_H_27_O_26_	935.0791	935.0824	633.0772, 300.9963, 275.0183, 249.0367
8.08	Ellagic acid-Pen	C_19_H_13_O_12_	433.0407	433.0378	300.9949, 275.0173, 249.0363

**Table 3:** The composition of the transfersomes loaded with the jabuticaba peel extract.

	Extract	S75	Tween80	Sodium Hyaluronate	Hydroxyethyl Cellulose
	mg/mL	mg/mL	mg/mL	mg/mL	mg/mL
Transfersomes	40	180	40		
1HEcellulose-transfersomes	40	180	40		1
2HEcellulose -transfersomes	40	180	40		2
1hyaluronan-transfersomes	40	180	40	1	
2hyaluronan-transfersomes	40	180	40	2	

**Table 4:** Mean diameter, polydispersity index, zeta potential and entrapment efficiency (EE) of transfersomes loaded with the extract obtained from the peel of jabuticaba fruits. Mean values ± standard deviations are reported (*n* = 6). Each symbol (*, °) indicates the same value that is different from that indicated by other symbols (*p* < 0.05).

	Mean Diameter (nm)	Polydispersity Index (PI)	Zeta Potential (mV)	EE (%)
Transfersomes	* 62 ± 4	0.23	−19 ± 2	90 ± 4
1IE-transfersomes	° 89 ± 2	0.25	−16 ± 4	90 ± 2
2IE-transfersomes	° 94 ± 5	0.21	−19 ± 2	94 ± 5
1IALO-transfersomes	° 89 ± 5	0.22	−18 ± 3	91 ± 2
2IALO-transfersomes	° 95 ± 4	0.25	−19 ± 2	95 ± 3

## Data Availability

Raw data are available and can be asked directly to authors.

## Sample Availability

Samples of the jaboticaba peel extract are available from the authors.
